# Viral shedding patterns of symptomatic SARS-CoV-2 infections by periods of variant predominance and vaccination status in Gyeonggi Province, Korea

**DOI:** 10.4178/epih.e2023008

**Published:** 2022-12-21

**Authors:** Gawon Choi, Ah-Young Lim, Sojin Choi, Kunhee Park, Soon Young Lee, Jong-Hun Kim

**Affiliations:** 1 Gyeonggi Infectious Disease Control Center, Health Bureau, Gyeonggi Provincial Government, Suwon, Korea; 2 Department of Social and Preventive Medicine, Sungkyunkwan University School of Medicine, Suwon, Korea; 3 Department of Preventive Medicine and Public Health, Ajou University School of Medicine, Suwon, Korea

**Keywords:** SARS-CoV-2, Viral load, Virus shedding, Variants, Vaccination

## Abstract

**OBJECTIVES:**

We compared the viral cycle threshold (Ct) values of infected patients to better understand viral kinetics by vaccination status during different periods of variant predominance in Gyeonggi Province, Korea.

**METHODS:**

We obtained case-specific data from the coronavirus disease 2019 (COVID-19) surveillance system, Gyeonggi in-depth epidemiological report system, and Health Insurance Review & Assessment Service from January 2020 to January 2022. We defined periods of variant predominance and explored Ct values by analyzing viral sequencing test results. Using a generalized additive model, we performed a nonlinear regression analysis to determine viral kinetics over time.

**RESULTS:**

Cases in the Delta variant’s period of predominance had higher viral shedding patterns than cases in other periods. The temporal change of viral shedding did not vary by vaccination status in the Omicron-predominant period, but viral shedding decreased in patients who had completed their third vaccination in the Delta-predominant period. During the Delta-predominant and Omicron-predominant periods, the time from symptom onset to peak viral shedding based on the E gene was approximately 2.4 days (95% confidence interval [CI], 2.2 to 2.5) and 2.1 days (95% CI, 2.0 to 2.1), respectively.

**CONCLUSIONS:**

In one-time tests conducted to diagnose COVID-19 in a large population, although no adjustment for individual characteristics was conducted, it was confirmed that viral shedding differed by the predominant strain and vaccination history. These results show the value of utilizing hundreds of thousands of test data produced at COVID-19 screening test centers.

## INTRODUCTION

Severe acute respiratory syndrome coronavirus 2 (SARS-CoV-2), first identified in Wuhan, China in winter 2019, has caused an ongoing pandemic. As of January 31, 2022, a total of 838,526 confirmed cases and 6,772 deaths (fatality rate, 0.78%) were recorded in Korea, despite strict social measures, vaccination campaigns, and therapeutic interventions [1].

SARS-CoV-2 infection has been confirmed by real-time reverse transcription polymerase chain reaction (RT-PCR) assay of respiratory tract samples, and RT-PCR results are shown as cycle threshold (Ct) values [2]. The Ct value itself does not indicate the amount of virus per sample, but by the well-known inverse correlation with viral load, temporal changes in Ct values have been used to understand the natural history of the virus [3,4] and to estimate viral shedding [5].

Many studies have shown that, like other respiratory viruses such as influenza and Middle East respiratory syndrome coronavirus (MERS-CoV), the Ct value of SARS-CoV-2 rapidly decreases after symptom onset, then gradually increases until a person tests negative [5]. However, the viral trajectories differ among factors such as mean peak Ct value, mean proliferative stage duration, clearance stage duration, and days from peak, and the Ct value peaks earlier in SARS-CoV-2 infections than for the 2003 severe acute respiratory syndrome or MERS-CoV [6]. Furthermore, factors such as individual characteristics (age, sex, underlying disease, and obesity) [3,7-9], variant types of SARS-CoV-2 [10-18], treatment, and vaccination history have affected the curve from beginning to end in periods of SARS-CoV-2 infection [19-22]. Thus, it is not clear exactly when viral shedding peaks and how much difference there may be in the viral load at the peak of a SARS-CoV-2 infection.

Viral load during SARS-CoV-2 infection also differs depending on the viral strain. For the ORF1ab target gene, the concentration of the Delta variant was 10 times higher than that of the wild type and 2 times higher than that of the Alpha variant [12]. Viral load affects levels of transmission and infectivity [5,22], and to reduce transmission, it is important to isolate the patient before viral shedding peaks, by analyzing the Ct value after symptom onset [6]. Viral load kinetics provide a basis for determining isolation duration, subsequent release timing, and any modifications to a standard isolation period according to the viral variant, the patient’s vaccination status, and resulting effects upon post-peak viral shedding [23]. In this study, we compared the Ct values of SARS-CoV-2 for confirmed patients across two years and evaluated viral shedding and effect of vaccination history during periods reflecting different variants’ local predominance.

## MATERIALS AND METHODS

### Data collection, criteria for exclusion

We obtained data about patients infected with coronavirus disease 2019 (COVID-19) through the COVID-19 surveillance system operated by the Korea Disease Control and Prevention Agency (KDCA), Gyeonggi in-depth epidemiological report system, and Health and Medical Crisis Response System operated by the Health Insurance & Review Assessment (HIRA) Service, from January 26, 2020 to January 31, 2022 in Gyeonggi Province, Korea [24,25]. We collected information on the confirmation date, whether symptoms were present at the time of diagnosis, and the date of symptom onset. In addition, we considered RT-PCR results including the Ct values of the E, RdRp, and N genes in the nasopharyngeal swab, the results of variant sequencing, and patients’ vaccination histories (e.g., date and type of vaccine).

The total number of patients identified by the COVID-19 surveillance system during the study period was 264,645. First, to observe temporal changes in viral Ct values since symptom onset, we excluded 49,617 cases (18.75%) that were asymptomatic or had an uncertain symptom onset. Then we extracted data suitable for each study condition among 215,028 cases for which the onset of symptoms was clearly confirmed. The Ct value was classified as positive if it was 35 or less according to laboratory criteria, and the remaining tests were classified as negative.

### Definition of dominant periods and vaccination status

**Figure 1.**
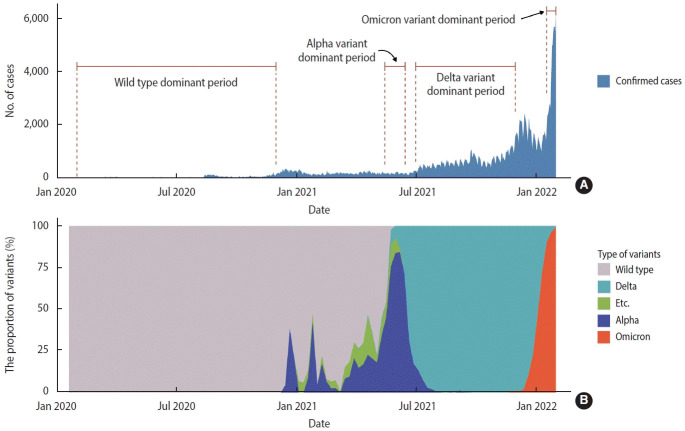
Trends in daily confirmed cases of severe acute respiratory syndrome coronavirus 2 (SARS-CoV-2) infection according to the period of SARS-CoV-2 variant predominance (A) and the proportion of virus type (B) in Gyeonggi Province, Korea from January 2020 to January 2022.

We designated specific dominant periods of SARS-CoV-2 variants that significantly affected the domestic epidemic situation. We defined these periods by when the proportion of a certain strain exceeded 50% of all variant-tested samples in Gyeonggi Province, Korea: wild type from February 1, 2020 to November 30, 2020, the Alpha variant from May 15, 2021 to June 15, 2021, the Delta variant from July 1, 2021 to November 30, 2021, and the Omicron variant from January 17, 2022 to January 31, 2022 (Figure 1).

Vaccination status was classified based on the number of vaccinations received and the time since vaccination. In this study, unvaccinated cases were individuals with confirmed cases who either had not been vaccinated or received 1 vaccine dose within 14 days prior to symptom onset. Moreover, those who were vaccinated with a second dose were defined as individuals with confirmed cases who received their second vaccine dose at least 14 days prior to symptom onset or their third dose within 14 days prior to symptom onset. Finally, those who were vaccinated with a third dose were defined as individuals whose third dose of vaccine had occurred more than 14 days before symptom onset. Our analysis excluded a first-dose-only group due to the small number of subjects.

### Statistical analysis

We presented categorical variables as numbers and proportions for confirmed patients according to the predominant periods of SARS-CoV-2 variants and vaccination status. The temporal Ct values were analyzed by fitting a generalized additive model (GAM) using R version 4.0.3 (R Foundation for Statistical Computing, Vienna, Austria):


Yt ~ Gaussian(µt)E(Y) = β0 + s(t)

where t refers to the day of observation, Yt refers to the Ct value observed at time t, s denotes a smoothing function, and time denotes the number of days since the onset of symptoms. Breakpoint analysis, which detects a change point in spline curves by fitting piecewise linear regressions, was used to estimate the peak viral load time and confidence interval (CI) [26,27].

### Ethics statement

This study was approved by the Institutional Review Board of Sungkyunkwan University School of Medicine (No. SKKU 2022-02-029). Informed consent was waived because all data were obtained as a result of a public health investigation.

## RESULTS

### The trend of SARS-CoV-2 cases by predominant periods of major variants

Wild-type SARS-CoV-2, identified in January 2020, prevailed as the predominant strain for about 17 months. Since the wild type was dominant for a long time, the period before the Alpha variant first appeared was considered to have been characterized by wild-type predominance (Figure 1A). The Alpha variant increased from December 2020 and became dominant from mid-May to mid-June 2021, while the Delta variant was identified starting in March 2021 and became the predominant strain for about 5 months. The Omicron variant began to increase from December 2021 and remained the predominant strain through the last week included in the study (Figure 1B).

### The number and proportion of confirmed cases in wild-type and variant periods of predominance

Among 215,154 cases available for analysis, the number of cases by period of predominance was 7,388 cases (3.4%) for wild type, 5,134 cases (2.4%) for Alpha, 90,830 cases (42.2%) for Delta, and 5,187 cases (2.4%) for Omicron (Table 1).

For the three types of target genes, we most often obtained the Ct value for the RdRp gene without omission. Although the number and proportion differed for each strain’s period of predominance, the proportion was the highest in the Alpha-predominant period at 99.2%, followed by the Delta-predominant period at 97.3%, the wild-type dominant period at 90.2%, and the Omicron-predominant period at 88.9%.

In confirmed cases, variant testing was performed in 4.8% of cases for the wild-type dominant period, 4.6% for the Alpha-predominant period, 10.5% for Delta, and 11.0% for Omicron. Wild-type virus was detected in all cases during wild-type predominance, and Alpha variants were detected in 69.2% of total cases during Alpha predominance. In the periods when Delta and Omicron dominated, 98.4% and 86.2% of the samples tested were Delta and Omicron variants, respectively.

No vaccination history was present during the wild-type period, and 99.3% of those infected during the Alpha-predominant period were unvaccinated. During the Delta-predominant period, 72.8% of patients were unvaccinated, and those who had completed their second and third doses accounted for 26.6% and 0.6%, respectively. During the Omicron-predominant period, the second and third dose completion rates were 45.0% and 23.0%. However, infected individuals who remained unvaccinated still accounted for a high proportion, 32.0%.

### Temporal changes in viral shedding by the specific period of variant predominance

**Figure 2.**
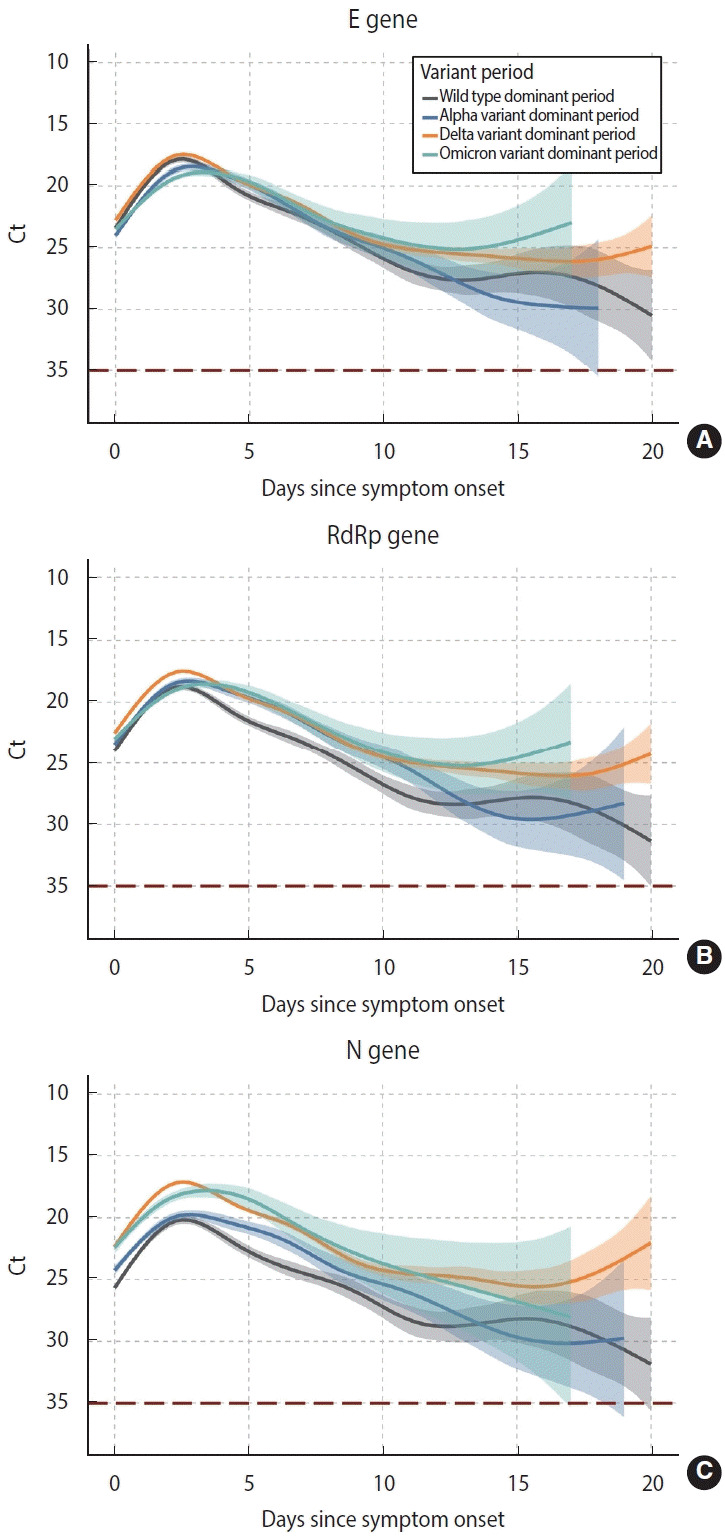
Comparison of temporal changes of viral shedding according to target genes: the E gene (A), RdRp gene (B), and N gene (C). Each target gene is compared by periods of variant predominance, and the thick curve shows the marginal effect of days since symptom onset of viral load with 95% confidence intervals from a generalized additive mixed model (shaded area). A negative polymerase chain reaction result was coded as a cycle threshold (Ct) value of 35.

Temporal changes in spline curves suggested that the Ct value, inversely correlated with viral load, peaked within 3 days after symptom onset and continuously decreased. Similar patterns were observed for the 3 target genes and 4 periods of variant predominance: E gene, RdRp gene, and N gene, and wild type, Alpha, Delta, and Omicron (Figure 2).

The peak time for the E gene was 2.1 days (95% CI, 2.0 to 2.3) during the wild-type dominant period, 2.3 days (95% CI, 2.2 to 2.5) during Alpha, 2.1 days (95% CI, 2.0 to 2.1) during Delta, and 2.4 days (95% CI, 2.2 to 2.5) during Omicron (Supplementary Material 1A). The peak time for the RdRp gene was 2.1 days in the wild-type and Alpha periods, and 2.4 days during Omicron (Supplementary Material 1B). For the N gene, it was 2.0 days during Delta and 2.4 days during Omicron (Supplementary Material 1C).

Viral shedding at peak during the Delta-predominant period was higher than for other strains during their periods of predominance, as found for all 3 target genes. Viral shedding during the Omicron-predominant period was lower and delayed compared to the Delta-predominant period (Figure 2).

### Changes in viral shedding pattern by vaccination status during periods of Delta and Omicron predominance

**Figure 3.**
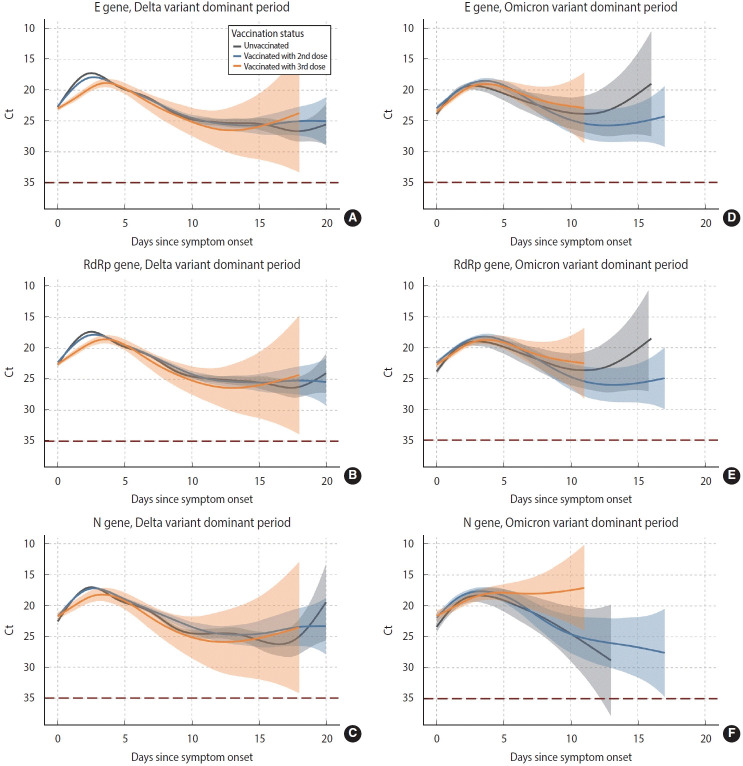
Temporal changes in the viral shedding pattern with vaccination status in the Delta (A-C) and Omicron (D-F) predominant periods. Each target gene (E gene: 2A and 2D, RdRp gene: 2B and 2E, and N gene: 2C and 2F) is compared by vaccination status. Ct, cycle threshold.

The peak and slope of viral shedding differed depending on vaccination status. During the Delta-predominant period, the peak of viral shedding was higher and earlier for the E gene in unvaccinated subjects, and lower and delayed in those who had completed their third dose. These findings were also observed in the RdRp and N genes (Figure 3A-C). In contrast, we found no differences in viral shedding in the Omicron-predominant period according to whether individuals were unvaccinated, vaccinated with a second dose, or vaccinated with a third dose (Figure 3D-F).

## DISCUSSION

We identified temporal changes in viral shedding by analyzing Ct values for SARS-CoV-2-infected patients in Gyeonggi Province, Korea across 2 years. As confirmed by many studies, regardless of whether an individual was infected with wild-type virus or a later variant, viral shedding increased after symptom onset and reached its peak within 3 days, then gradually decreased. In addition, we materialized the peak time of viral shedding and compared it according to each major strain’s predominant period. The peak time was the shortest during the Delta-predominant period (2.0-2.1 days) and longest during the Omicron-predominant period (2.4 days) for all 3 target genes (E, RdRp, and N).

The peak time of viral shedding has been reported as 2-4 days for wild-type SARS-CoV-2 and 1-3 days for the Delta variant [3,4,14,28]. The Omicron variant’s peak time was also reported to be 2-5 days after symptom onset, and a Japanese study found that the peak time was delayed compared to other variants [29,30]. In this study on the peak time of viral shedding, Ct values for symptomatic individuals were gathered from serial tests or a one-time test at each infected individual’s time of diagnosis. Although administering consecutive tests per individual is a more objective method that recognizes the natural progression of viral shedding, we analyzed the data obtained by correlating the Ct value at the time of diagnosis with the date of symptom onset, rather than repeated measurements of a set of infected individuals. Nevertheless, we confirmed that our results were similar to those of the consecutive test method [3,28].

**Figure 4.**
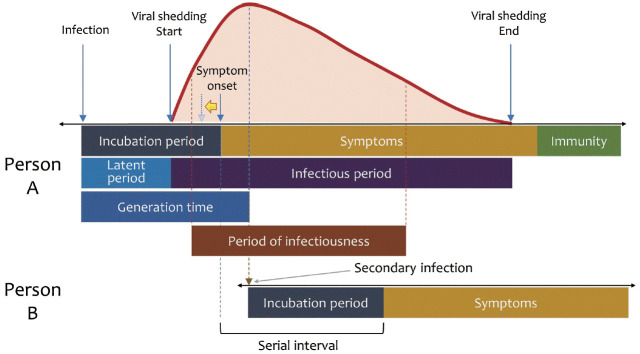
Schematic of the relationship between person A and person B in the transmission of severe acute respiratory syndrome coronavirus 2. The yellow arrow indicates a time shift in which the symptom onset date is shifted to the left in cases infected with Omicron, as opposed to cases infected with the previous variants, resulting in a seemingly delayed peak from symptom onset.

The incubation and serial interval periods of individuals infected with Omicron were reduced compared to those with Delta [31-33]. According to Figure 4, as the incubation period is shortened, symptom onset moves toward the yellow arrow. Consequently, the time from symptom onset to the peak is lengthened, as in the Japanese study cited above [30]. Therefore, it is more appropriate to interpret the delayed Ct peak observed during the Omicron-predominant period in our study as a result of earlier symptom onset after infection, rather than a longer time to peak after infection.

In periods of infection, the generation time minus the incubation period corresponds to the time from symptom onset to viral peak in our study (Figure 4). The generation time and the incubation period of wild-type virus were 6.2 days and about 3 days [34], whereas for the Delta variant, the respective values were 6.84-3.59 days (depending on the strength of the contact) and 3-4 days [33,35], which supports the approximately 2-day time from symptom onset to viral peak confirmed in our study. We observed that the time from symptom onset to peak was 2.2-2.3 days for the Alpha-predominant period and 2.0-2.1 days for the Delta-predominant period, or about 9% shorter during Alpha than Delta. It supports findings by Hart et al. [36] that the Delta variant’s generation time was about 28% shorter than that of Alpha.

In a retrospective analysis using the average Ct value, an increase in the ratio of low Ct values was accompanied by an explosion of confirmed patients. This may have been affected by the increased transmissibility of a high viral load, or it could also be a warning about the emergence of new variants where conducting viral sequencing tests for all infectors is impossible [37]. The Alpha, Delta, and Omicron variants showed increased viral load compared to wild type [11,12,18,22], and Delta was higher than Alpha [14], but there was no significant difference between Delta and Omicron [15-17].

Viral shedding has been studied for its correlations with several variables. Age and sex did not show differences between patients infected with the Wuhan strain and Delta [5,38]. However, there was a correlation between viral shedding and the grade of disease severity. High viral shedding observed early in the course of disease may indicate aggravated clinical disease [3], and in another study of the Wuhan strain, patients with more than moderate symptoms showed higher viral shedding at the peak compared to those with mild symptoms or asymptomatic patients [11,13,39]. We did not analyze viral shedding according to severity, but the high viral load during our study’s Delta-predominant period might explain symptom severity in patients infected with the Delta variant [40].

Underlying diseases that lower immunity, such as malignancies and acquired immune deficiency syndrome (AIDS), can prolong viral shedding [7-9]. In the case of lymphoma patients, the virus was excreted from the upper respiratory tract for up to 2 months, and the median duration was observed to be 30 days and 22 days in AIDS patients and solid cancer patients, respectively. In addition, several COVID-19 treatments have been used with emergency approval so far, and Remdesivir (GS-5734) and Regkirona (CT-P59), which were introduced early, might have a complex effect on viral shedding from the wild-type and Alpha variants, respectively. In our study, we could not confirm a difference in the duration of viral shedding according to pre-existing disease, nor a change in viral shedding according to the type of treatment. Nevertheless, studies of viral shedding obtained by one-time tests of the time interval from symptom onset to diagnosis are highly significant [14,28].

The vaccination campaign against SARS-CoV-2 began 13 months after the first confirmed case in Korea. On February 26, 2021, in addition to the adenovirus-vector based Oxford/AstraZeneca (ChAdOx1) (AstraZeneca) vaccine, the two-dose messenger RNA (mRNA) vaccines BNT162b2 (Pfizer–BioNTech) and mRNA-1273 (Moderna) were introduced, and as of January 31, 2022, the third, second, and first doses of vaccination rate had reached 53.1%, 85.7%, and 87.0% of the population, respectively [1]. Clinically, vaccination showed an effect of 92-95% in preventing symptomatic infection and severe disease [41,42], as well as a reduction in infectiousness and disease mortality [43-45]. Vaccination lowered the viral concentration during infection [19,20]. Although a study found no difference in the viral load peak according to vaccination, it mentioned the limitations of a small sample size and not representing the general population [15]. Despite these different opinions, Delta’s viral load decreased faster in vaccinated patients than unvaccinated patients [21,46]. We observed that peak viral shedding was low in third-dose recipients during the Delta-predominant period, but viral clearance according to vaccination could not be confirmed. However, we also observed that during the Delta-predominant period, viral shedding patterns did not differ significantly between the second-dose vaccinated group and the unvaccinated group, whereas these patterns differed from that of the third-dose vaccinated group. In our study, the viral shedding of those infected with Omicron did not differ by vaccination status. Similar results also have been identified in some studies [15,47], although a reduced infectious viral load was observed in boosted Omicron infectors [22].

This study has several limitations. First, as mentioned before, this study analyzed the test results of many subjects at the time of COVID-19 confirmation. In general, however, consecutive measurements after symptom onset in the same individual represent the gold standard for evaluating viral shedding. Therefore, these results may differ from the results of consecutive tests for confirmed individuals with COVID-19. Second, the time interval from vaccination to infection was not considered in the analytical model. This may cause differences in results, even if we take vaccination status into account. Third, since the SARS-CoV-2 confirmation tests were conducted using different products, there could have been differences in the Ct value results depending on the product’s characteristics, but this was not evaluated. Nevertheless, this study systematically organized primary data on SARS-CoV-2 shedding patterns based on more than 100,000 test data for the estimated SARS-CoV-2 strains dominant in Korea. Since new SARS-CoV-2 strains are expected to appear in the future, understanding the epidemiological characteristics of existing viruses will help interpret the variability of COVID-19 epidemiology and provide effective management of infected individuals.

## Figures and Tables

**Table 1.** The number and proportion of available data for target genes, variant testing, and vaccination status by local periods of SARS-CoV-2 variant predominance

Available data		Predominance period of specific strain			
		Wild type (n=7,388)	Alpha variant (n=5,134)	Delta variant (n=90,830)	Omicron variant (n=5,187)
Target genes					
	E gene	6,652 (90.0)	4,433 (86.4)	81,501 (89.7)	4,340 (83.7)
	RdRp gene	6,666 (90.2)	5,095 (99.2)	88,405 (97.3)	4,611 (88.9)
	N gene	4,055 (54.9)	3,274 (63.8)	42,640 (46.9)	1,660 (32.0)
Variant testing		357 (4.8)	237 (4.6)	9,532 (10.5)	572 (11.0)
	Wild type	357 (100)	39 (16.5)	0 (0.0)	0 (0.0)
	Alpha	0 (0.0)	164 (69.2)	152 (1.6)	0 (0.0)
	Delta	0 (0.0)	16 (6.8)	9,375 (98.4)	79 (13.8)
	Omicron	0 (0.0)	0 (0.0)	2 (0.0)	493 (86.2)
	Other	0 (0.0)	18 (7.6)	3 (0.0)	0 (0.0)
Vaccination status^1^		n=7,388	n=5,038	n=81,719	n=5,023
	Unvaccinated	7,388 (100)	5,004 (99.3)	59,528 (72.8)	1,604 (32.0)
	Second dose complete	0 (0.0)	34 (0.7)	21,776 (26.6)	2,262 (45.0)
	Third dose complete	0 (0.0)	0 (0.0)	415 (0.6)	1,157 (23.0)

Values are presented as number (%). SARS-CoV-2, severe acute respiratory syndrome coronavirus 2.

1 First vaccination dose was excluded.

## SUPPLEMENTARY MATERIALS

Supplementary Material 1A

Supplementary Material 1B

Supplementary Material 1C

## References

[1] https://www.kdca.go.kr/filepath/boardSyview.es?bid=0015&list_no=718518&seq=2(Korean).

[2] Corman VM, Landt O, Kaiser M, Molenkamp R, Meijer A, Chu DK (2020). Detection of 2019 novel coronavirus (2019-nCoV) by real-time RT-PCR. Euro Surveill.

[3] Lim AY, Cheong HK, Oh YJ, Lee JK, So JB, Kim HJ (2021). Modeling the early temporal dynamics of viral load in respiratory tract specimens of COVID-19 patients in Incheon, the Republic of Korea. Int J Infect Dis.

[4] Walsh KA, Jordan K, Clyne B, Rohde D, Drummond L, Byrne P (2020). SARS-CoV-2 detection, viral load and infectivity over the course of an infection. J Infect.

[5] He X, Lau EH, Wu P, Deng X, Wang J, Hao X (2020). Temporal dynamics in viral shedding and transmissibility of COVID-19. Nat Med.

[6] Cevik M, Tate M, Lloyd O, Maraolo AE, Schafers J, Ho A (2021). SARS-CoV-2, SARS-CoV, and MERS-CoV viral load dynamics, duration of viral shedding, and infectiousness: a systematic review and meta-analysis. Lancet Microbe.

[7] Niyonkuru M, Pedersen RM, Assing K, Andersen TE, Skov MN, Johansen IS (2021). Prolonged viral shedding of SARS-CoV-2 in two immunocompromised patients, a case report. BMC Infect Dis.

[8] Huang J, Xie N, Hu X, Yan H, Ding J, Liu P (2021). Epidemiological, virological and serological features of coronavirus disease 2019 (COVID-19) cases in people living with human immunodeficiency virus in Wuhan: a population-based cohort study. Clin Infect Dis.

[9] Rogado J, Gullón P, Obispo B, Serrano G, Lara MÁ (2021). Prolonged SARS-CoV-2 viral shedding in patients with solid tumours and associated factors. Eur J Cancer.

[10] Acer Ö, Genç Bahçe Y, Özüdoğru O (2022). Association of viral load with age, gender, disease severity, and death in severe acute respiratory syndrome coronavirus 2 variants. J Med Virol.

[11] Wang Y, Chen R, Hu F, Lan Y, Yang Z, Zhan C (2021). Transmission, viral kinetics and clinical characteristics of the emergent SARS-CoV-2 Delta VOC in Guangzhou, China. EClinicalMedicine.

[12] Teyssou E, Delagrèverie H, Visseaux B, Lambert-Niclot S, Brichler S, Ferre V (2021). The Delta SARS-CoV-2 variant has a higher viral load than the Beta and the historical variants in nasopharyngeal samples from newly diagnosed COVID-19 patients. J Infect.

[13] Ong SW, Chiew CJ, Ang LW, Mak TM, Cui L, Toh MP (2022). Clinical and virological features of severe acute respiratory syndrome coronavirus 2 (SARS-CoV-2) variants of concern: a retrospective cohort study comparing B.1.1.7 (Alpha), B.1.351 (Beta), and B.1.617.2 (Delta). Clin Infect Dis.

[14] Kang M, Xin H, Yuan J, Ali ST, Liang Z, Zhang J (2022). Transmission dynamics and epidemiological characteristics of SARS-CoV-2 Delta variant infections in Guangdong, China, May to June 2021. Euro Surveill.

[15] Migueres M, Dimeglio C, Trémeaux P, Abravanel F, Raymond S, Lhomme S (2022). Influence of immune escape and nasopharyngeal virus load on the spread of SARS-CoV-2 Omicron variant. J Infect.

[16] Hay JA, Kissler SM, Fauver JR, Mack C, Tai CG, Samant RM (2022). Quantifying the impact of immune history and variant on SARS-CoV-2 viral kinetics and infection rebound: a retrospective cohort study. Elife.

[17] Cedro-Tanda A, Gómez-Romero L, de Anda-Jauregui G, GarnicaLópez D, Alfaro-Mora Y, Sánchez-Xochipa S (2022). Early genomic, epidemiological, and clinical description of the SARS-CoV-2 Omicron variant in Mexico City. Viruses.

[18] Julin CH, Robertson AH, Hungnes O, Tunheim G, Bekkevold T, Laake I (2021). Household transmission of SARS-CoV-2: a prospective longitudinal study showing higher viral load and increased transmissibility of the alpha variant compared to previous strains. Microorganisms.

[19] Levine-Tiefenbrun M, Yelin I, Alapi H, Katz R, Herzel E, Kuint J (2021). Viral loads of Delta-variant SARS-CoV-2 breakthrough infections after vaccination and booster with BNT162b2. Nat Med.

[20] Eyre DW, Taylor D, Purver M, Chapman D, Fowler T, Pouwels KB (2022). Effect of Covid-19 vaccination on transmission of alpha and delta variants. N Engl J Med.

[21] Singanayagam A, Hakki S, Dunning J, Madon KJ, Crone MA, Koycheva A (2022). Community transmission and viral load kinetics of the SARS-CoV-2 delta (B.1.617.2) variant in vaccinated and unvaccinated individuals in the UK: a prospective, longitudinal, cohort study. Lancet Infect Dis.

[22] Puhach O, Adea K, Hulo N, Sattonnet P, Genecand C, Iten A (2022). Infectious viral load in unvaccinated and vaccinated individuals infected with ancestral, Delta or Omicron SARS-CoV-2. Nat Med.

[23] https://www.who.int/news-room/commentaries/detail/criteria-for-releasing-covid-19-patients-from-isolation.

[24] https://covid19.kdca.go.kr.

[25] https://hcr.hira.or.kr/hira_hcr/index.jsp.

[26] Muggeo VM (2003). Estimating regression models with unknown breakpoints. Stat Med.

[27] Muggeo VM (2017). Interval estimation for the breakpoint in segmented regression: a smoothed score‐based approach. Aust N Z J Stat.

[28] Kim JM, Rhee JE, Yoo M, Kim HM, Lee NJ, Woo SH (2022). Increase in viral load in patients with SARS-CoV-2 delta variant infection in the Republic of Korea. Front Microbiol.

[29] Takahashi K, Ishikane M, Ujiie M, Iwamoto N, Okumura N, Sato T (2022). Duration of infectious virus shedding by SARS-CoV-2 Omicron variant-infected vaccinees. Emerg Infect Dis.

[30] Torjesen I (2022). Covid-19: peak of viral shedding is later with omicron variant, Japanese data suggest. BMJ.

[31] Backer JA, Eggink D, Andeweg SP, Veldhuijzen IK, van Maarseveen N, Vermaas K (2022). Shorter serial intervals in SARS-CoV-2 cases with Omicron BA.1 variant compared with Delta variant, the Netherlands, 13 to 26 December 2021. Euro Surveill.

[32] Del Águila-Mejía J, Wallmann R, Calvo-Montes J, RodríguezLozano J, Valle-Madrazo T, Aginagalde-Llorente A (2022). Secondary attack rate, transmission and incubation periods, and serial interval of SARS-CoV-2 Omicron variant, Spain. Emerg Infect Dis.

[33] Jansen L, Tegomoh B, Lange K, Showalter K, Figliomeni J, Abdalhamid B (2021). Investigation of a SARS-CoV-2 B.1.1.529 (Omicron) variant cluster - Nebraska, November-December 2021. MMWR Morb Mortal Wkly Rep.

[34] Ki M, Task Force for 2019-nCoV (2020). Epidemiologic characteristics of early cases with 2019 novel coronavirus (2019-nCoV) disease in Korea. Epidemiol Health.

[35] Manica M, De Bellis A, Guzzetta G, Mancuso P, Vicentini M, Venturelli F (2022). Intrinsic generation time of the SARS-CoV-2 Omicron variant: an observational study of household transmission. Lancet Reg Health Eur.

[36] Hart WS, Miller E, Andrews NJ, Waight P, Maini PK, Funk S (2022). Generation time of the alpha and delta SARS-CoV-2 variants: an epidemiological analysis. Lancet Infect Dis.

[37] Mishra B, Ranjan J, Purushotham P, Saha S, Payal P, Kar P (2022). High proportion of low cycle threshold value as an early indicator of COVID-19 surge. J Med Virol.

[38] Hwang H, Lim JS, Song SA, Achangwa C, Sim W, Kim G (2022). Transmission dynamics of the Delta variant of SARS-CoV-2 infections in South Korea. J Infect Dis.

[39] Stankiewicz Karita HC, Dong TQ, Johnston C, Neuzil KM, PaascheOrlow MK, Kissinger PJ (2022). Trajectory of viral RNA load among persons with incident SARS-CoV-2 G614 infection (Wuhan strain) in association with COVID-19 symptom onset and severity. JAMA Netw Open.

[40] Lauring AS, Tenforde MW, Chappell JD, Gaglani M, Ginde AA, McNeal T (2022). Clinical severity of, and effectiveness of mRNA vaccines against, covid-19 from omicron, delta, and alpha SARS-CoV-2 variants in the United States: prospective observational study. BMJ.

[41] Angel Y, Spitzer A, Henig O, Saiag E, Sprecher E, Padova H (2021). Association between vaccination with BNT162b2 and incidence of symptomatic and asymptomatic SARS-CoV-2 infections among health care workers. JAMA.

[42] Baden LR, El Sahly HM, Essink B, Kotloff K, Frey S, Novak R (2021). Efficacy and safety of the mRNA-1273 SARS-CoV-2 vaccine. N Engl J Med.

[43] Haas EJ, Angulo FJ, McLaughlin JM, Anis E, Singer SR, Khan F (2021). Impact and effectiveness of mRNA BNT162b2 vaccine against SARS-CoV-2 infections and COVID-19 cases, hospitalisations, and deaths following a nationwide vaccination campaign in Israel: an observational study using national surveillance data. Lancet.

[44] Pritchard E, Matthews PC, Stoesser N, Eyre DW, Gethings O, Vihta KD (2021). Impact of vaccination on new SARS-CoV-2 infections in the United Kingdom. Nat Med.

[45] Vasileiou E, Simpson CR, Shi T, Kerr S, Agrawal U, Akbari A (2021). Interim findings from first-dose mass COVID-19 vaccination roll-out and COVID-19 hospital admissions in Scotland: a national prospective cohort study. Lancet.

[46] Chia PY, Ong SW, Chiew CJ, Ang LW, Chavatte JM, Mak TM (2022). Virological and serological kinetics of SARS-CoV-2 Delta variant vaccine breakthrough infections: a multicentre cohort study. Clin Microbiol Infect.

[47] Fall A, Eldesouki RE, Sachithanandham J, Morris CP, Norton JM, Gaston DC (2022). The displacement of the SARS-CoV-2 variant Delta with Omicron: an investigation of hospital admissions and upper respiratory viral loads. EBioMedicine.

